# Prevalence of reduced respiratory muscle strength in institutionalized elderly people

**DOI:** 10.1590/S1516-31802009000200005

**Published:** 2009-07-06

**Authors:** Rodrigo Polaquini Simões, Viviane Castello, Marco Antonio Auad, Jadiane Dionísio, Marisa Mazzonetto

**Affiliations:** 1 Physiotherapist. Postgraduate student in the Postgraduate Program on Physiotherapy (Cardiopulmonary Physiotherapy Laboratory), Universidade Federal de São Carlos (UFSCar), São Carlos, São Paulo, Brazil.; 2 Physiotherapist. Postgraduate student in the Postgraduate Program on Biotechnology, Universidade Federal de São Carlos (UFSCar), São Carlos, São Paulo, Brazil.; 3 Physiotherapist. Postgraduate student in the Postgraduate Program on Physiotherapy, Universidade Federal de São Carlos (UFSCar), São Carlos, São Paulo, Brazil.; 4 Physiotherapist. Professor in the Department of Physiotherapy, Universidade Camilo Castelo Branco (Unicastelo), Descalvado, São Paulo, Brazil.

**Keywords:** Respiratory system, Respiratory muscles, Muscle strength, Homes for the aged, Aged., Sistema respiratório, Músculos respiratórios, Força muscular, Instituição de longa permanência para idosos, Idoso.

## Abstract

**CONTEXT AND OBJECTIVES:**

Respiratory muscle strength is relevant to the clinical situation of elderly patients, particularly those presenting with respiratory or cardiac diseases. The objectives of this study were to evaluate the respiratory muscle strength of institutionalized elderly women, compare this with predicted values for the Brazilian population and calculate the correlation with age and anthropometric characteristics.

**DESIGN AND SETTING:**

Cross-sectional study at the Department of Physiotherapy of Universidade Camilo Castelo Branco.

**METHODS:**

The participants were 56 institutionalized elderly women (74.87 ± 10.55 years of age), evaluated in eight institutions in three cities in the central region of the State of São Paulo, between January 2005 and March 2006. They were separated into three subgroups according to age: 60-69 years (n = 20), 70-79 (n = 18) and 80-89 years (n = 18). Maximal respiratory pressures were obtained using a manovacuometer. The values obtained were compared between subgroups and with predicted values. Correlation analysis was used to evaluate age, weight, height and body mass index in relation to maximal respiratory pressures. The significance level was P < 0.05.

**RESULTS:**

No significant differences in maximal respiratory pressures were seen between the three subgroups. The maximal respiratory pressures were significantly lower in the three subgroups, compared with predicted values. Negative correlations between maximal respiratory pressures and age and positive correlations in relation to weight, height and body mass index were found.

**CONCLUSIONS:**

Respiratory muscle strength was markedly reduced in institutionalized 60 to 89-year-old women and the values demonstrated correlations with age and anthropometric characteristics.

**CLINICAL TRIAL REGISTRATION NUMBER:**

ACTRN12608000136303

## RESUMO

**CONTEXTO E OBJETIVOS:**

A força muscular respiratória é relevante para a situação clínica de pacientes idosos, particularmente os que apresentam doença cardíaca ou respiratória. Os objetivos deste estudo foram avaliar a força muscular respiratória de mulheres idosas asiladas, comparar com os valores preditos para a população brasileira e correlacioná-los com a idade e características antropométricas.

**TIPO DE ESTUDO E LOCAL:**

Estudo transversal desenvolvido pelo Departamento de Fisioterapia da Universidade Camilo Castelo Branco.

**MÉTODOS:**

Foram estudadas 56 mulheres idosas asiladas (74,87 ± 10,55 anos) sendo avaliadas em oito instituições em três cidades do interior do estado de São Paulo, entre janeiro de 2005 e março de 2006. Elas foram separadas em três subgrupos: de 60-69 anos (n = 20), de 70-79 anos (n = 18) e de 80-89 anos (n = 18). As pressões respiratórias máximas foram obtidas através de um manovacuômetro. Os valores obtidos foram comparados entre os subgrupos e também com os preditos para a população brasileira. Análise de correlação foi aplicada para a idade, peso, altura e índice de massa corpórea com os valores das pressões respiratórias máximas. O nível de significância adotado foi P < 0,05.

**RESULTADOS:**

Não houve diferenças significativas das pressões respiratórias máximas entre os três subgrupos. Em relação aos valores preditos, as pressões respiratórias máximas obtidas foram significativamente menores nos três subgrupos. Correlações negativas entre as pressões respiratórias máximas e a idade e positivas em relação ao peso, altura e índice de massa corpórea foram encontradas.

**CONCLUSÕES:**

A força muscular respiratória é marcantemente reduzida em idosas asiladas de 60 a 89 anos, havendo correlação das pressões respiratórias máximas com a idade e com as características antropométricas.

## INTRODUCTION

Life expectancy is increasing all over the world and the number of people aged 60 years or over in Brazil had increased to 19 million by 2006 (10.2% of the total population). The elderly population in Brazil is likely to reach more than 30 million by the end of the next 20 years, a number that would represent 13% of the population.^1^

Increasing demand for long-term care institutions^2^ (residential institutions) has accompanied this accelerated demographic transition in Brazil. The principal factors that lead to institutionalization of the elderly have been observed with greater frequency over the last three years. These factors are precarious social support, low income and the burden of increased spending for medical and health needs.^3^

Research has shown that the institutionalized elderly have a higher incidence of health complications in general,^4^ and this has become an important public health problem because of the consequent increased demand for services.^5^ It has also been shown that hospital stays are longer for these elderly people^6^ and that the percentages of complications and mortality are higher than among non-institutionalized elderly individuals,^5^ since the most common reasons for hospitalization are trauma, fractures and neurological, cardiovascular and respiratory diseases.^7^^,^^8^

Respiratory diseases occur with greater frequency when the functioning of the musculature responsible for the respiratory system is impaired.^9^^,^^10^^,^^11^ Respiratory muscle dysfunction can lead to reduced tolerance for the demands of activities of daily living, and to hyperventilation and, in extreme cases, respiratory failure. Reduced respiratory muscle strength is relevant to the clinical situation of elderly patients, particularly in cases of pneumonia and cardiac diseases such as left ventricle failure, which lead to additional workload for the respiratory muscles.^12^

Evaluation of respiratory muscle strength is of great clinical importance^13^ and it can be done by measuring static and dynamic maneuvers. Static maneuvers infer the respiratory muscle strength through measurement of the sub and supra-atmospheric pressure generated by inspiratory and expiratory muscles, respectively. These pressures evaluated through the mouth reflect the pressure that is being generated in the alveolus by the action of respiratory muscles.^14^^,^^15^^,^^16^

Maximal respiratory pressure measurement using a manovacuometer is a simple and effective technique. Its accuracy has already been proven in the literature in relation to its ability to identify changes in these respiratory pressures. It has been in continual use since the 1960s and 1970s because of its important diagnostic and prognostic role in evaluating both healthy individuals and patients with certain chronic diseases.^14^^,^^17^^,^^18^^,^^19^

Regarding the accuracy and reliability of such equipment, Hamnegård et al.^20^ compared measurements using a manovacuometer with measurements using a pressure transducer (special equipment for measuring pressures that is used as a reference) and did not find any significant differences, thereby showing that the manovacuometer measurements were accurate and reproducible. In a study on elderly subjects, McConnell et al.^21^ found that the coefficient of variation was 10.2% for maximal inspiratory pressure and 12.8% for maximal expiratory pressure, thus showing good reproducibility. Moreover, it has been reported that respiratory muscle strength is linked to the level of physical effort during activities of daily living.^14^^,^^22^^,^^23^

Studies^14^^,^^15^ have been carried out with the intention of establishing normal values for respiratory muscle strength in relation to the Brazilian population. The most pertinent study to date is that of Neder et al.,^14^ in which the authors proposed predictive formulae for calculating maximal inspiratory pressure and maximal expiratory pressure.

Based on these reports, we hypothesized that the maximal respiratory pressure among the institutionalized elderly would be lower than the normal values predicted for the Brazilian population. In addition, we speculated that individual anthropometric values would have a relationship with maximal respiratory pressures.

## OBJECTIVES

The objectives of this study were to evaluate respiratory muscle strength through measurements of maximal inspiratory and expiratory pressures, among institutionalized women from 60 to 89 years of age, and to compare these data with predicted values for the Brazilian population. Furthermore, the study aimed to correlate maximal respiratory pressure values with age, weight, height and body mass index.

## METHODS

### Design

This was a cross-sectional study on institutionalized elderly women. The data were gathered from eight institutions in neighboring cities in the central region of the interior of the State of São Paulo (three institutions in the city of São Carlos, three in Descalvado and two in Araraquara), between January 2005 and March 2006.

### Subjects

The selection process in each institution took approximately two weeks. Firstly, the women were preselected according to age, which was taken from their medical records in their institution. After this preselection, they underwent an initial evaluation and only the women who fulfilled the inclusion criteria were selected for this study.

The participants selected for this study were women aged over 60 years, with body mass index classification of eutrophic (body mass index from 18.5 to 24.9 kg/m^2^) or overweight (body mass index from 25 to 29.9 kg/m^2^), in accordance with the World Health Organization^24^ criteria. They were not involved in regular physical activity, had no prior history of cardiovascular or respiratory diseases, and had never been smokers. They also did not present any abnormalities in the thoracic or abdominal regions, such as cutaneous retractions or accentuated structural deviations in the spinal column that might alter the respiratory dynamics.

Initially, 74 institutionalized elderly women were evaluated. Out of this total, 18 were not included in the sample: seven presented difficulties in performing the maneuvers adequately; four were former smokers; three presented body mass index greater than 30 kg/m^2^; two presented lateral spinal deviation; one had a history of partial lobectomy of the right lung; and one backed out during the initial evaluation.

Thus, this study involved 56 elderly women who had been institutionalized for approximately 8 ± 3 years (mean ± standard deviation), of ages ranging from 60 to 89 years (74.87 ± 10.55 years). These subjects were separated into three subgroups according to age: 60 to 69 years (n = 20), 70 to 79 years (n = 18) and 80 to 89 years (n = 18). The ages and anthropometric characteristics of each subgroup are presented in Table 1.

**Table 1.** Study subjects’ ages and anthropometric characteristics

	Age (years)	Weight (kg)	Height (m)	BMI (kg/m^2^)
60-69 years	64.22 ± 3.28	64.28 ± 7.76	1.63 ± 0.06	24.08 ± 1.48
70-79 years	75.06 ± 2.98	62.17 ± 7.61	1.60 ± 0.06	24.14 ± 1.88
80-89 years	85.33 ± 2.89	59.06 ± 7.12	1.58 ± 0.07	23.61 ± 1.24

Data are reported as means and standard deviations. BMI = body mass index.

### Ethical matters

All procedures were explained and described in detail in a consent statement that was signed by all participants and by the persons responsible for their respective long-term care in these institutions, in accordance with Resolution 196/96 of the Brazilian National Health Council and with the ethical principals stated in the World Medical Association’s Helsinki Declaration. Additionally, this research was reviewed and approved by the Ethics Committee for Human Research of our Institution.

Variables evaluated

#### Initial evaluation

After identifying the elderly subjects aged over 60 years (preselection), through information contained in their medical records, they underwent an initial evaluation. This consisted of anamnesis: personal data such as full name, age, diet and occupation performed in the past; medical history such as cardiovascular and respiratory diseases, previous surgery and possible use of medications; and whether they smoked or had previously smoked.

#### Anthropometry and physical evaluation

A physical evaluation was conducted to identify possible abnormalities in the thoracic or abdominal regions, such as cutaneous retractions or accentuated structural deviations in the spinal column that might alter the respiratory dynamics.

Weight was measured on a previously calibrated balance (Welmy, São Paulo, SP, Brazil), and a stadiometer forming part of this same equipment (Welmy) was used to measure height. The latter was done after the individual took a deep inspiration while standing erect. The subjects were barefoot and wore lightweight clothing during these measurements. The body mass index was calculated from the weight and height measurements in order to classify the nutritional status (body mass index = weight/height^2^; kg/m^2^).

#### Respiratory muscle strength

An aneroid manovacuometer (GERAR, São Paulo, SP, Brazil) with an operational range of ± 300 cmH_2_O was used to obtain maximal inspiratory and expiratory pressure values. A tube was connected to the apparatus and its distal extremity was connected to a plastic device with an orifice of approximately 2 mm in diameter.^17^ This allowed small-scale air leakage, in order to avoid any elevation of pressure in the oral cavity that might be generated by the facial musculature.^18^ To cover the plastic device, a mouthpiece of 32 mm in diameter was provided for each individual.

Before measurements were taken, each individual was seated and familiarized with the equipment and shown how the maneuvers were to be done. The maximal inspiratory and expiratory pressures maneuvers were carried out for a minimum of three trials with an interval of one minute between each repetition. Ten seconds before the maneuver was performed, a nasal clip was placed on the individual (to prevent air from escaping through the nostrils) and then, with the lips tightly closed, the forced inspiratory maneuver was performed from the residual volume or the forced expiratory maneuver was performed from the total pulmonary capacity. The maximal respiratory effort was sustained for approximately one second.^14^

The highest value of three correctly accomplished repetitions (difference of 10% or less between trial values) was recorded for each trial.^14^ The manovacuometer measurements for all individuals were carried out by a single researcher, using the same verbal commands in all cases.

After the maximal inspiratory and expiratory pressures measurements had been collected, comparisons with predicted values for the Brazilian population were made. The predicted values for each individual were calculated in accordance with the formula of Neder et al.,^14^ as follows:

maximal inspiratory pressure = -0.49 (age) + 110.5

and

maximal expiratory pressure = -0.81 (age) + 115.7.

#### Data analysis

The evaluations were done by three experienced researchers: one responsible for preselection and anamnesis; another researcher for physical and anthropometric evaluations, and a third researcher responsible for collecting the maximal respiratory pressures. Additionally, for analysis of the results, one researcher who participated in the data gathering also helped in the data analysis, together with two other researchers who were solely responsible for examining the data. We obtained test-retest coefficients of variation (CV) for maximal inspiratory and expiratory pressures for eighteen individuals, and the values were 8.2% for inspiratory pressure and 9.6% for expiratory pressure.

#### Statistical analysis

The Kolmogorov-Smirnov test was used to investigate the data distribution, and it confirmed that the distribution was normal. The data were then expressed as means and standard deviations. The sample size was calculated using the GraphPad StatMate software, version 1.01. To reach statistical significance (P < 0.05 at a power of 80% with a confidence interval of 95%) a sample of 16 subjects in each of the three subgroups was needed in order to show a 20% difference in maximal inspiratory and expiratory pressures. The magnitudes of expected differences were derived from previous investigations.^14^^,^^15^ This total sample was thus 56 women (two groups with 18 and one group with 20 women.

Analysis of variance (ANOVA) for independent groups was used to compare values between subgroups. If significant differences were found, the Tukey-Kramer post-hoc test was used to identify these differences. Student’s t-test was applied to compare the values obtained with the predicted values. The Pearson correlation analysis was used to evaluate the correlation levels of age, weight, height and body mass index in relation to the maximal respiratory pressure values. The probability of type I error occurrence was established at 5% for all tests (a = 0.05). The data were analyzed using the Statistica for Windows software (Stat Soft Inc, 2000).

## RESULTS

No significant differences were found when the maximal inspiratory and expiratory pressures values were compared for the three age subgroups (maximal inspiratory pressure, P = 0.07 and maximal expiratory pressure, P = 0.31), as shown in Table 2. Significantly lower values (P < 0.001) for the three subgroups were found for maximal respiratory pressures in relation to the predicted values (Table 2).

Maximal respiratory pressures presented a moderate negative correlation with age and a moderate positive correlation with body mass index. There was a strong positive correlation in relation to weight and a moderate positive correlation for height in relation to maximal expiratory pressure and a strong correlation in relation to maximal inspiratory pressure (Table 3).

**Table 2.** Comparison of maximal respiratory pressures between obtained and predicted values for each subgroup and among subgroups

	MIP (O) cmH_2_O	MIP (P) cmH_2_O	MEP (O) cmH_2_O	MEP (P) cmH_2_O
60-69 years	44.44 ± 15.33	79.03 ± 1.61*	41.94 ± 14.96	63.68 ± 2.66*
70-79 years	37.50 ± 12.40	73.72 ± 1.46*	37.50 ± 14.88	54.91 ± 2.41*
80-89 years	33.06 ± 16.10	68.69 ± 1.42*	34.78 ± 12.11	46.58 ± 2.34*

MIP = maximal inspiratory pressure; MEP = maximal expiratory pressure; (O) = obtained; (P) = predicted. *Significant difference between predicted and obtained values (P < 0.001). There was no significant differ ence among subgroups.

**Table 3.** Correlation between maximal respiratory pressures and anthropometric characteristics

	MIP (cmH_2_O)			MEP (cmH_2_O)		
	r	CI	P-value	r	CI	P-value
Age (years)	-0.56	-0.42 - -0.68	0.03	-0.52	-0.40 - -0.71	0.03
Weight (kg)	0.94	0.78 - 0.97	0.01	0.80	0.67 - 0.91	0.01
Height (m)	0.87	0.65 - 0.92	0.01	0.68	0.52 - 0.80	0.03
BMI (kg/m^2^)	0.68	0.50 - 0.83	0.02	0.66	0.55 - 0.78	0.02

r = correlation value; CI = 95% confidence interval; MIP = maximal inspiratory pressure; MEP = maximal expiratory pressure; BMI = body mass index.

## DISCUSSION

Considering the significant increase in the population of elderly people in Brazil^25^ and the expected increase in demand for residential long-term care institutions in the future,^2^ evaluations on respiratory muscle strength among institutionalized elderly people are of great importance. It needs to be borne in mind that impairment of respiratory muscle strength is responsible for respiratory complications that worsen the health problems among institutionalized elderly people.

The main findings from this study reveal that the respiratory muscle strength of these institutionalized elderly people was markedly reduced in relation to the normal values established for the Brazilian population. There was also a strong relationship between anthropometric data and respiratory muscle strength.

Despite the finding of a moderate negative correlation between age and maximal respiratory pressures (r = -0.56 for maximal inspiratory pressure and r = -0.52 for maximal expiratory pressure, i.e. an inverse relationship between respiratory muscle strength and increasing age), no significant differences in maximal inspiratory and maximal expiratory pressure were found between the different age subgroups (Table 2). This suggests that the strength levels of inspiratory and expiratory musculature were not modified by aging in this population. These findings do not corroborate the results from the majority of published studies, which have indeed reported decreasing respiratory muscle strength with aging subgroups. However, these other studies were not conducted among institutionalized elderly populations.^14^^,^^26^^,^^27^

In a recent study by our research group, Simões et al.^26^ evaluated 100 healthy individuals between 40 and 89 years of age and found that there was a significant and progressive diminution of respiratory muscle strength with aging. They reported that the possible explanations for this result were connected with the physiological modifications that are caused by the aging process itself. These include changes to the pulmonary tissue and rib cage, calcification of rib articulation cartilage, loss of muscle mass (a process known as sarcopenia) in the diaphragm and accessory musculature, and lower muscle response to neural stimulation. However, one possible explanation for our findings of no differences in respiratory muscle strength between the subgroups may be that the individuals in our study had already been institutionalized for a relatively long period of time (8 ± 3 years). Over this period, they may have already reached the maximal impairment and thus showed little or no further change.

This possible impairment of respiratory muscle strength in the elderly women evaluated is supported by the significantly lower maximal inspiratory and expiratory pressures of our findings, in comparison with the predicted values for the Brazilian population, as proposed in the literature and particularly by Neder et al.^14^. Respiratory muscle strength impairment can be linked directly to the lower level of physical activity in the daily routines of institutionalized elderly individuals. They spend a great deal of their time sitting and lying down, and they only walk short distances, in contrast with the greater activity of non-institutionalized elderly people. This inactivity accelerates the process of sarcopenia, which probably occurs in the peripheral muscles as well as the respiratory musculature.

The relationship between physical activity and muscle strength has been described in the literature by Power and Criswell,^28^ who reported that increased muscle strength and endurance capacity is positively associated with higher levels of physical activity. Several studies^14^^,^^21^^,^^29^^,^^30^ have shown that physical inactivity promotes peripheral muscle loss as well as decreases in respiratory muscle strength. Neder et al.^14^ reported that there was an association between peripheral muscle force (as measured using the knee extensor muscles) and respiratory muscle strength (as measured using maximal respiratory pressure), since individuals with lower extensor muscle strength also presented lower values for maximal respiratory pressures. There also appears to be a relationship between upper limb force and respiratory muscle strength, since a study by Enright et al.^29^ found a positive correlation between handgrip strength and respiratory muscle strength in elderly subjects.

There was a strong positive correlation between weight measurements and maximal respiratory pressures, i.e. the greater the body weight was, the greater the respiratory muscle strength was. This finding is concordant with the study by Schoenberg et al.^31^ The latter reported that weight exerted a certain influence on respiratory muscle strength, because as body weight increased, the volume and size of respiratory muscles increased, which improved the strength of these muscles and ventilatory function. However, it must be noted that the individuals evaluated in the present study were eutrophic or overweight. Therefore, despite finding a strong correlation, the characteristics of the sample selected should be considered with caution, because other studies have proven that obese individuals gradually show decreasing respiratory muscle strength as they gain weight.

Some authors^32^^,^^33^^,^^34^^,^^35^ have reported that the reduction in respiratory muscle strength in obese subjects in their studies was probably caused by reductions in thoracic compliance. The adipose tissue deposited in the thorax and abdomen of obese individuals promotes mechanical compression of the thoracic cavity, which leads to decreased compliance.^33^^,^^34^^,^^35^ This increase in the mechanical resistance of the thoracic wall causes greater elastic resistance, which makes expansion of the thorax during inspiration more difficult and consequently impairs full compliance of the respiratory system.^36^^,^^37^^,^^38^

A strong positive correlation was found between height and maximal inspiratory pressure, and a moderate positive correlation was found with maximal expiratory pressure values. This therefore infers that the taller the elderly individual is, the greater the respiratory muscle strength is, and it may reflect the relationship between pulmonary volume and maximal respiratory pressures. It has been reported^39^ that pulmonary volume has a direct relationship with height, considering that the highest values of maximal inspiratory and expiratory pressures are generated with the smallest and largest pulmonary volumes, respectively. It can be inferred that maximal respiratory pressures are dependent on pulmonary volume and, consequently, the latter are dependent on the individual’s height.

Moderate positive correlations were found between body mass index and maximal respiratory pressures. These results suggest that the greater the body mass index is in elderly women, the greater their respiratory muscle strength is. However, it needs to be noted that this relationship between weight and height does not take into account the quantities of lean and fat mass in individuals. Therefore, caution must be used when inferring that respiratory muscle strength is lower or higher in relation to body mass index. Nonetheless, body mass index is widely utilized to correlate the degree of obesity^32^^,^^36^^,^^40^ with the respiratory muscle strength in obese populations. The great majority of studies^32^^,^^36^ conducted among obese individuals have revealed an inverse relationship between body mass index and respiratory muscle strength, i.e. the greater their body mass index is, the lower their respiratory muscle strength is. According to Naimark and Cherniack,^36^ depending on the degree to which body mass index increases in obese individuals, respiratory muscle strength may decline significantly because of the reduction in compliance of the rib cage, due to accumulation of fat mass in this region.

This prevalence study was able to identify the weakness of the respiratory muscles in these institutionalized elderly women. It emphasizes the need to implement interventions relating to respiratory muscle strength in this population, thereby promoting improvements relating to respiratory function and preventing complications that directly relate to reductions in this strength.

With regard to limitations of this study, it should be noted that the subjects did not undergo spirometric tests. These would have provided information of greater precision on their respiratory clinical conditions. Nor was there any test to determine the lean and fat mass. Lack of data on lean and fat mass limited our ability to make greater inferences in relation to body composition and its possible relationship with respiratory muscle strength.

## CONCLUSIONS

Our data allows us to conclude that respiratory muscle strength is markedly reduced in institutionalized elderly women between the ages of 60 and 89 years, and that there is a relationship between anthropometric characteristics and the level of respiratory muscle strength.
